# An Effective and Secure Key Management Protocol for Message Delivery in Autonomous Vehicular Clouds

**DOI:** 10.3390/s18092896

**Published:** 2018-08-31

**Authors:** Congcong Li, Shouwen Ji, Xi Zhang, Haiping Wang, Dongfeng Li, Huiyong Liu

**Affiliations:** 1 School of Traffic and Transportation, Beijing Jiaotong University, Haidian District, Beijing 100000, China; 14114206@bjtu.edu.cn (C.L.); xizhang@bjtu.edu.cn (X.Z.); 15114198@bjtu.edu.cn (H.L.); 2 Department of Research and Development, Beijing Zhonghaiwenda Information Technology Company, Haidian District, Beijing 100000, China; whp@mail.ustc.edu.cn; 3 Electronic Transaction Cryptographic Application Group, State Cryptography Administration Office of Security Commercial Code Administration, Fengtai District, Beijing 100000, China; lidongfeng@refinepay.com

**Keywords:** group key management, authentication, certificateless, Chinese Reminder Theorem, vehicular cloud

## Abstract

Autonomous vehicular clouds, as the combination of cloud computing and conventional vehicular ad hoc networks, will provide abundant resources and services by sharing under-utilized resources of future high-end vehicles such as computing power, storage and internet connectivity. Autonomous vehicular clouds will have significant impact if widely implemented in the intelligent transportation system. However, security and privacy issues are still big challenges in autonomous vehicular clouds. In this paper, after analyzing the particularity of autonomous vehicular clouds, we implement a two-layered architecture, in which vehicles are self-organized without the help of roadside units. Then based on the architecture, we put forward an effective key management protocol to distribute a group key efficiently and also provide the authentication and confidentiality that lots of current secure schemes ignore. In addition, according to the different scenarios and security levels we categorize the way of message transmitting into three kinds. At last, with performance evaluations, the proposed protocol can perform more efficiently than other well-known available schemes.

## 1. Introduction

With the wide usage of intelligent transportation system (ITS) and the variety of wireless communication technologies, vehicular ad-hoc networks (VANETs), as the important part of ITS, have become of paramount importance in modern traffic management. VANETs provide different services for drivers and allow them to share sensitive traffic information with other drivers such as accident avoidance warnings, weather conditions and state of vehicles, which can improve traffic management efficiency and safety.

However, with the increased shared traffic information and the more types of applications users need such as in-vehicle multimedia entertainment, vehicular social networking and location-based services, a single vehicle has limited computation and storage resources, which leads to low data processing capability [1].

Recently, a few studies have proposed the concept of vehicular cloud computing (VCC) that combines cloud and VANET. VCC is a new paradigm that has a prominent impact on traffic management and road safety [2] and it has been developed to overcome the drawbacks in VANET. Nowadays, VCC is considered as the key measure to improve and develop ITS [3].

Oraliu et al. [4] first proposed the concept of autonomous vehicular clouds (AVCs). In AVCs, a number of vehicles with abundant resources are viewed as service providers [5]. This is a group of largely autonomous vehicles that contribute their computing, sensing, communication and physical resources to the cloud. Vehicles’ resources and the information exchanged from the vehicles with the cloud can be used by other vehicles in decision making [6]. There are two modes in AVCs, zero-infrastructure vehicular clouds and infrastructure-based vehicular clouds. The first mode can provide more stable communication structure due to the high mobility of highway vehicle nodes, which leads to the short of network communication time and the loss of allocated resources. With this in mind, this paper is mainly focused on the zero-infrastructure autonomous vehicular clouds, which few researchers have studied.

With the wide use of AVCs, the significance of security is also on the rise. A more careful analysis reveals that many of the classic security challenges are exacerbated by the characteristic features of AVCs [7]. In addition, it is more complex to distribute keys of vehicles due to the characteristic of zero-infrastructure vehicular clouds. Therefore, an efficient and secure key management protocol, which can manage active groups and provide an authentication as well as confidentiality with less computation and communication overhead, is necessary.

In the study of AVCs, most studies only focus on the security challenges [2,3,7,8], the architecture and traffic flow control [1,9,10,11,12,13], but in the literature, just a few studies have addressed a specific solution to these security challenges in AVCs. Furthermore, among these existing protocols in the active network, most of them have addressed authentication but fail to propose an authentication as well as confidentiality protocol. In this paper, according to the threats in AVCs, we propose an efficient and secure key distribution protocol to ensure the secure communication and the confidentiality of the traffic information in AVCs. The main contributions provided are as follows:

The proposed key management protocol is based on the Chinese Reminder Theory (CRT) and certificateless public key cryptograph (CLPKC) [14], which supports a high secure and efficient authentication and confidentiality in AVCs. The major advantages of the protocol are that updating keys during the users’ join and leave operations is performed efficiently. Besides, it solves the certificate management problem in the public key infrastructure (PKI) [15]. With performance evaluations, this proposed protocol gets a promising result from a better trade-off between efficiency and security than other current schemes discussed in the literature. This protocol is considered as a better adaptation in AVCs.

Due to the high mobility of vehicular nodes in the zero-infrastructure scenario, we design a two-layered architecture, in which vehicles self-organize to groups to ensure the better communication stability. Furthermore, in order to reduce the computation overhead at a vehicle side and a cloud server side, the proposed protocol categorizes the traffic information and the way of information transmission into three types, respectively. These features allow our protocol to be performed in various scenarios.

We perform some experiments to measure the energy consumption of the proposed protocol. The experimental results show that our protocol can handle subgroup key management more efficiently than other well-known schemes. Our scheme is lightweight and hence more suitable for AVCs.

The remainder of this paper is organized as follows: Section 2 summarizes the previous works in the literature. The problem statement and the system model are presented in Section 3. We describe our proposed key management protocol in Section 4 and the security analysis about the protocol in Section 5. Section 6 provides the performance evaluation and results of our proposed algorithm with the other current schemes. Section 7 gives a simulator for the proposed protocol. Section 8 gives concluding remarks and suggests some future directions.

## 2. Related Work

In this section, we provide a brief summary of the related literature addressed the security issues in AVCs and some related popular group key management schemes in VANETs. Yu et al. [1] in 2013 proposed an infrastructure-based architecture for VCs, which included a vehicular cloud, a roadside cloud, and a central cloud. Then they studied cloud resource allocation and virtual machine migration in this cloud-based vehicular network. Hussain et al. [11] in 2014 introduced a multi-layered architecture for VCs and proposed a context-aware dynamic parking service based on the cloud-assisted architecture. Hussain [13] in 2012 proposed a three-architecture framework including vehicular clouds, vehicles using clouds (VuC), and hybrid vehicular clouds (HVC). Ahmed [3] in 2018 proposed a novel privacy-preserving mechanism for VCs. In this paper, the authors focus on the architecture and highlight applications and features in VCs. Also, they explain the challenges for VCs. Finally, the authors present opportunities and future for VCs. However, these aforementioned researches only focused on providing the concept and the architecture for VCs, but the issues of security and privacy have not yet been addressed in the literature.

Aloqaily [16] proposed a quality of experience (QoE) software architecture in vehicular clouds, where QoE requirements are collected via numerous vehicular nodes in the vehicular cloud and re-formulated by a weighted combination of some important factors such as revealed to the Trusted Third Party (TTP). Through this scheme, users can select the best appropriate TTP and service providers (SPs) depending on the QoE-aware values. However, this architecture is multicenter-based, it has to be ensuring the number of TTPs between the users and SPs. The difficulty is to ensure the credible identity of TTPs and to protect from collusion attacks.

Aloqaily, also [17] introduced a scheme that provides continuous vehicular services by predicting the future location of vehicles. This scheme used QoE methodology to provide appropriate services among service providers. However, computing prescient location for vehicles and providing services ahead of time are more time-consuming than providing services using real-time data from sensor nodes.

Sánchez-García [18] in 2015 proposed a secure authentication scheme with applications for urban VANET scenarios using the Spanish eID, which allows authorities to easily and automatically obtain drivers’ identities. However, the communication is only from the road authority vehicles (e.g., police vehicles) to other vehicles for requesting a driver to be authenticated in case it is suspected of any road infraction. In addition, the PKI-based authentication mechanisms require additional computation overhead to verify the certificates of others.

Rajeshwari [7] in 2016 discussed that security and privacy are the two major challenges in AVCs and it is divided into three subsets in this paper: security threats, security requirements, and security challenges. The paper classified the architecture of AVCs into three layers: networks, the wireless communication channel and cloud computing. Also, it considered that PKI is one of the most important key management in AVCs. However, PKI will have problems in certification management and keys revocation in AVCs. 

Wang [19] in 2016 presented a secure and privacy-preserving navigation scheme for fog-based VANETs. This scheme based on a message-locked encryption (MLE) and randomized convergent encryption (RCE) supports authentication, confidentiality and conditional privacy preservation. However, this scheme only accounts for the location matching without considering content matching. The scheme leads to additional communication and computational costs, because that the user will decrypt all the returned cipher texts included in the route and has to find the point of interest.

Otoum [20] in 2018 proposed an adaptively supervised and intrusion-aware data aggregation for wireless sensor clusters in critical infrastructures. The proposed scheme undertook the intrusion problem by using adaptation strategy to detect dynamically known and unknown intrusions. And it demonstrated that the proposed method performs with 99% detection rate and 99.80% accuracy in the presence of known and unknown malicious behavior in the WSN. However, it only focuses on the detection strategy without active defense strategy. 

According to threats in AVCs, some literature proposed related methods to ensure the security in AVCs. The secure protocols listed below are based on the certification.

Yan et al. [7] in 2013 analyzed a variety of security challenges and potential privacy threats that are specific to VCs. Then this paper proposed a geographic location-based security mechanism to ensure security for VCs. The scheme divides the city map into a number of cells with a virtual machine. However, the vehicle could not decrypt the messages if it is not in the decryption region. Therefore, the scheme has a limited scalability of communication in VCs. Also, the scheme is based on the PKI, which may cause an additional communication overhead for managing certifications. At last, the key revocation of the scheme is using certificate revocation lists (CRLs), which will occupies the large amounts of storage.

Lin et al. [21] in 2008 proposed a timed efficient and secure vehicular communication (TSVC) scheme using hash chains to protect the privacy of users. In this scheme, it utilizes the group signature without helping of infrastructure to verify messages. Due to the short message authentication code (MAC) attached to each packet, the communication and computation overhead are significantly reduced compared with other current public key based authentication schemes. However, this scheme is based on the certification and each vehicle is preloaded with many anonymous public/private key pairs and their corresponding public key certificates at the initialization stage, which occupies more storage of vehicles than other certificationless schemes. Moreover, when vehicle members frequently change from a group, the leader vehicle of the group broadcasts its hash chain much more frequently, which will induce the increased message loss ratio. Also, the scheme fails to propose a confidentiality services for users.

Because of the certificate management problem, some other schemes proposed ID based authentication for vehicular-network applications.

Abdulrahman et al. [5] in 2017 proposed a lightweight privacy-preserving scheme, which provided confidentiality and integrity in AVCs. This scheme supports RSU-based architecture and the RSU, acts as the proxy ID, resigncrypts the messages from a certificate authority (CA) server However, because of the high mobility of vehicles in AVCs, it is time-consuming for RSUs to resigncrypt messages. Besides, the information on the scheme is one-way transmission from the CA server to vehicles and it does not support vehicle-to-vehicle communication. Furthermore, the CA server generates the secret key for every user and hence it cannot resist insider attacks. Finally, this method is based on bilinear pairing and the relative computation overhead of a pairing operation is approximately 20 times higher than that of an elliptic curve scalar multiplication [22].

Chim [23] in 2011 proposed an ID-based authentication scheme based on the bilinear pairings with lower communication costs for secure V-to-I communications. However, Horng et al. [22] in 2013 found that Chim’s scheme was vulnerable to impersonation attacks. Horng et al. [24] provided a secure scheme that can achieve the security and privacy requirements, and overcame the weaknesses of the scheme in [23]. However, the computation overhead of one pairing operations is at least three times higher than that of a one point multiplication operation [25]. Hence these schemes based on bilinear pairings are not suitable for rapidly changing networks. Also, these schemes cannot ensure the confidentiality of messages, which could not support privacy messages communication. 

Park [26] in 2018 proposed a trajectory-based message delivery for cloud-assisted VANETs. The vehicles can share their trajectory messages to specified socialspot roadside units through the center. The strategy adopted a certificateless proxy re-encryption (CL-PRE) scheme and an ID-based signature (ID-SIG) scheme, with which it can get better security for user’s privacy messages. However, this scheme fails to support the conditional privacy preservation. The TA cannot trace malicious vehicles and obtain their real identities, which are not stored in the TA to protect from table-stealing attacks. Furthermore, in the phase of trajectory sharing on the cloud, when one vehicle send the message $Tm_{d}=\left\{{\mathrm{pid}}_{d0}{,C,\mathrm{ts},S}_{d}\right\}$, and ${\mathrm{id}}_{d0}$, a unchanged PID, is easy for malicious vehicles to link messages from the same vehicle. In addition, the relative computation overhead of a pairing operation is higher than that of an elliptic curve scalar multiplication. 

He et al. [27] in 2015 proposed an ID-based scheme using the Elliptic Curve Cryptography (ECC), which was more efficient than previously proposed authentication schemes. However, this scheme heavily relies on a tamper-proof hardware device. Lo et al. [28] in 2016 proposed a faster ID-based scheme based on ECC and the scheme supported batch verification. However, it is significantly dependent on secure communication and it has high costs and limited scalability. Also, these two schemes are only focus on authenticity but the confidentiality.

Yang [29] in 2018 proposed an authentication scheme based on certificateless signature with message recovery. The scheme supports conditional privacy, batch verification and unlinkability in VNENTs. The evaluation results indicate that the scheme achieves low computation and communication costs without the bilinear pairing and map-to-point hash operations. However, this scheme is a one-way communication only from vehicles to RSUs, where vehicles cannot access the traffic information sent from the center. This transmission mechanism affects the function of information sharing in VENETs.

In the following, we will discuss some of the current group key management methods, which not only support integrity but confidentiality.

Lim [30] in 2016 proposed an efficient group signature scheme based on certification in VANETs. Each RSU acts as a leader of one group, is responsible for the distribution and updating of pseudo identities (IDs), original IDs, certificates and shared secret keys. However, according to the driving speed of individual vehicles, especially in the highway scenario, the distance between one mobile vehicle and a static RSU is changed quickly. Therefore, it is time consuming to initiate the key establishment process frequently when the vehicle enters a communication region covered by a new RSU. Furthermore, in this infrastructure-based architecture, if a vehicle wants to download some huge documents, it will fail to finish downloading when moving out from the communication range of the RSU. Also, the scheme does not provide the group key updating process when one member leaves or joins a group, which is not supporting the backward and forward secrecy. When one vehicle leaves or joins the region of a RSU, it can also access the information from the RSU using the group key $K_{G_{i}}$.

Vijayakumawe [31] in 2016 proposed a dual group key management scheme for VANETs. In this scheme, the vehicle users are categorized into three groups, which have different authorities to access the service. With the distribution and updating of a group key via Chinese Reminder Theorem (CRT), this scheme can get a more computationally efficient result compared with other related schemes. However, the information transmission is in a one-way manner, in which the scheme does not refer to how Secondary Users (SUs) or Primary Users (PUs) delivers messages to the TA. Furthermore, it is not efficient for message transmission. If there is no PUs around SUs, SUs could not obtain traffic information in time. In addition, with the group secrete key of SUs, PUs can learn about any of information of SUs. At last, the scheme is lacking of privacy preservation. When the PUs broadcast the message to SUs by $E_{k_{\mathrm{sug}}}\left({\mathrm{ID}}_{v}∥\mathrm{message}\right)∥E_{\mathrm{TA}-\mathrm{Pvt}}\left(\mathrm{AC}∥{\mathrm{ID}}_{v}∥{\mathrm{TS}}_{3}∥\mathrm{Lifetime}\right)$, the identity of this PU ${\mathrm{ID}}_{v}$ is exposed to the public.

Zheng et al. [32] in 2007 proposed two centralized group key management protocols (CRGK and FCRGK) based on the CRT. The proposed approach minimizes the storage complexity at the TA side and the number of broadcast messages at the user side. However, the computation complexity is high at the key distribution aspect.

Zhou [33] in 2009 designed a group key distribution algorithm based on the CRT. The main advantages are that it reduces the key server’s computation complexity of the group key distribution and when the number of group members increase to a certain number, the computing time of the key server will decrease. However, this also increases the workload of finding the root IDs in the group member subtrees and a common group key by congruent equations.

Seo [34] in 2015 proposed a certificateless-effective key management (CL-EKM) protocol in dynamic wireless sensor networks (WSNs). It is noted that it can significantly reduce the communication overhead by CL-EKM based on the cluster structure. This scheme supports efficient key updating when a node leaves or joins a cluster and ensures forward and backward key secrecy. However, the method of updating cluster key is more time consuming. The group leader informs every member of new keys by pairwise session keys of its cluster.

Compared with the current authentication and group key management schemes mentioned in the literature, in this paper, we propose a key management protocol that is superior to others in many ways. First, the protocol is based on the certificateless public key cryptograph (CLPKC) using ECC, which can solve the certificate management problem in the PKI and the key escrow problem in the identity-based encryption (IBE) with lower computational overhead. Furthermore, the protocol provides authenticity plus confidentiality for communication among vehicles. Moreover, according to the different security levels we categorize keys into four kinds and traffic information into three kinds. In order to minimize the computation overhead of key distribution and management, we use the CRT-based key management approach. With performance evaluations, the computational cost in the proposed protocol is lower than other related schemes.

## 3. Proposed Vehicular Networks Architecture

### 3.1. Problem Statement and Solution Objectives

#### 3.1.1. Time-Consumption

We consider two scenarios here. For urban roads in a city block, a number of vehicles are prone to co-located when a traffic-related event or traffic jam has occurred, which leads to a low average speed of vehicles. Multiple vehicles which observe the same phenomena will send repetitive traffic-related messages to the cloud sever which can result in the increased burden for the cloud server. Therefore, the messages should be analyzed, verified and discarded if it redundant before sending to the cloud server.

#### 3.1.2. Cost-Effectiveness

On highways, vehicles are distributed non-uniformly, and the high mobility of vehicles leads to short-time communication in the network. Therefore, the communication range between mobile vehicles and fixed infrastructure such as RSUs, acting as gateway to link vehicles and cloud servers, is limited. Furthermore, the RSUs are deployed in the highways, unattended environments, so the cost of constructing infrastructure is high. From an implementation perspective, the designed protocol should ensure the stability of the communication quality and the low cost high efficiency.

#### 3.1.3. Verification 

When a vehicle encounters problems such as accidents, bad road conditions due to bad weather or traffic jams, etc., it needs to propagate that information to other nearby vehicles and the transportation centers in appropriate regions. If an adversary inserts malicious messages or modifies messages to the network, this will lead to traffic chaos or even accidents. Therefore, the authentication of the messages should be ensured.

#### 3.1.4. Anonymity

The anonymity of drivers should be preserved to protect the identities of drivers from being revealed by adversaries. Also, the information sent from the vehicles should not be traced by other vehicles.

#### 3.1.5. Confidentiality

Some information about privacy of drivers should be protected from being eavesdropped. Thus, the confidentiality of messages should be ensured.

#### 3.1.6. Light Weight

Due to the high mobility of vehicles and the limited computation power of on board units (OBUs), a lightweight key management protocol with fast authentication and encryption/decryption should be designed.

In this paper, we present an efficient key management protocol, which addresses and solves the aforementioned problems.

### 3.2. Cloud Model

According to the two scenarios in city blocks and highways mentioned above, we adopt a zero-infrastructure with two-layer architecture to better suitable for the AVCs. The two-layered architecture includes the autonomous vehicular cloud (AVC) and the central cloud (CC):

- *Autonomous Vehicular Cloud*: A mobile cloud consists of a large number of mobile sensor nodes played by autonomous vehicles and a set of aggregation-and-forwarding nodes, namely subgroup leaders (GLs). Each GL aggregates the data from the other vehicle nodes in its subgroup and forwards to the CC. The vehicles provide under-utilized resources and in return they receive the welfare from the central cloud for services [35]. Vehicles in the vicinal communication range select the GL by the method [36] which acts as the gateways. All vehicles in VCs are divided into many subgroups that are administered by their respective GLs. The vehicles can access the resources from cloud servers by vehicle to cloud communication (V2C) and other vehicles by vehicle to vehicle (V2V) communication. As a self-organized group of cooperative vehicles, they can lengthen the communication durability within its own communication range. The decentralized architecture can make less cost of the central cloud and reach an effective implement and key delivery.
- *Central Cloud*: A central cloud has many more resources and is mainly used for registration, complicated computation, massive data storage, and global decisions [1]. The central cloud provides services for vehicles. Here, mobile edge computing (MEC) implemented at the base stations (BS) is useful in reducing latency and improving localized user experience [37]. Delay-sensitive services are executed and delivered much faster due to the close proximity of end users to the MEC infrastructure [38].
- *Private Key Generator* (*PKG*): The PKG included in the CC is a trusted party that generates partial private keys to users. The PKG is fully trusted by all parties in the network and cannot be compromised.

This cloud architecture has several essential advantages. First, energy-sufficient vehicles can act as ideal active candidates to provide their storage, computational ability and sensing capabilities on the move. Second, this architecture is especially adapted to the highway scenario, which can cut down the expenditure of building fixed infrastructure. Furthermore, the average speed of vehicles is high. The vehicles form a subgroup to avoid communication breakage and it is helpful to enlarge the communication range when accessing the data by V2V rather than the fixed infrastructure, so the self-organized group structure is beneficial for the continuity and reliability of accessing cloud services. Last but not least, it is more convenient and secure for the CC and GLs to manage secret keys. Further details on the protocol and security proof are elaborated in the next section.

## 4. Proposed Protocol

In this section, we present an effective and secure key management protocol for autonomous vehicle clouds. In order to improve the security and reduce the energy consumption of communication, the proposed protocol categorizes the transmitting ways into three kinds according to the different security levels. There are four kinds of keys for different message delivery: a group key r within the range of all authorized vehicular nodes, a subgroup key *GK*, a secret key $d_{2}$ between the PKG and a vehicle, and a secret key k between a group leader and a vehicle.

### 4.1. Types of Keys

The functions of the different types of keys are shown in Table 1.

- *The Secret Key between the PKG and a Vehicle*: Each vehicle shares a unique individual key $d_{2}$ with the PKG. For example, if a vehicle wants to transmit the sensitive data to the CC directly bypassing the GL, or if it fails to communicate with the GLs, the node can encrypt the information using the secret key and send it to the CC. In addition, it is convenient for the CC to calculate the group key via $d_{2}$.
- *Group Key*: We consider all the authorized vehicles as one large group, in which all registered vehicles share one secret key. The group key is mainly used for broadcasting traffic messages without obtaining by other illegal vehicles, and also it can exclude the compromised node from the group easily through changing the group key. Only the CC can update the key when a vehicle leaves or joins the group.
- *Subgroup Key*: In the cluster-based structure, the pool of members is categorized into small groups called subgroups, which can minimize the computation complexity in the key updating phase and achieve a secure key delivery. All vehicles in a subgroup share one key, named subgroup key, which is mainly used for securing broadcast messages, such as sensitive commands or the change of member status in one subgroup. Only the GL can update the subgroup key when the member joins or leaves the subgroup.
- *Secret Key between the GL and the Vehicle*: The function of constructing the secret key between the GL and the vehicle is to calculate the subgroup key for them. It is a secure way to deliver the secret key k by key agreement instead of generating it by the GL itself.

### 4.2. Types of Information

In this section, the proposed protocol divides the information into three types:

- *Public traffic information*: This kind of traffic information collected by vehicular nodes and analyzed by the central cloud aims to help vehicles take intelligent decisions and enhance security and quality of driving. These traffic messages based on related services and applications include real time traffic information, intelligent decision hint, localizing events and emergency warning etc.
- *Private custom information*: This type of information is related to the privacy of drivers, such as parking space booking, infotainment, electronic logging, service spot detection, specific route navigation and some other sensitive data. The different service requirements should be encrypted to protect from being eavesdropped.
- *Group sharing information*: This type of information used to be shared with all members in one subgroup, such as the private change of member status, updating subgroup keys and subgroup services etc. This kind of information should be acquired by the subgroup members rather than others.

The different kinds of information are summarized in Table 2.

### 4.3. Types of Transmitting

**Figure 1:**
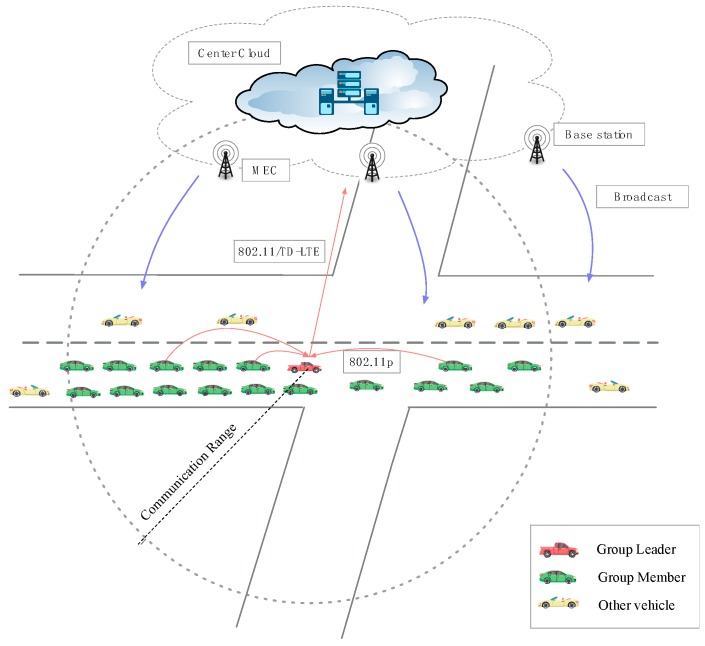
Transmission path of Public traffic information.

**Figure 2:**
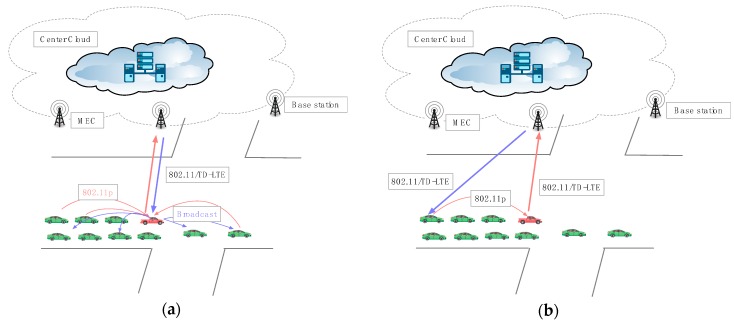
Two kinds of transmission path: (**a**) Subgroup-sharing information; (**b**) Private custom information.

In order to strengthen the security of information communication in the autonomous network, this proposed protocol considers three ways of information transmission according to the different security level of information as shown in Figure 1 and Figure 2.

In the first scenario (seen in Figure 1), the vehicles share the public traffic information in real time. The difference from other protocols is that the GL classifies and filters the messages after receiving them from subgroup members and then transfers them to the central cloud. With the help of the framework based on the efficient vertical-handover operation (VHO) and service-level-agreements (SLAs) scheme [39], the mobile nodes, especially GLs, can access different networks with stable communication and low service delay. 

The benefit is that this can reduce the redundant and repetitive traffic messages and achieve cost reductions, especially in intersections or the spot of an accident where a lot of vehicles are co-locating for the red light or traffic events. Then, the central clouds broadcast the messages to the authorized members directly.

The second scenario (seen in Figure 2a) shows that the GL sends the requirement to the central cloud without being known by other vehicles during the subgroup key updating process. Then the central cloud feeds back the related information to the GL.

In the third scenario (seen in Figure 2b), the GL transfers the private custom information to the CC, and then it returns the service information to the vehicle directly through the end-to-end way bypass the GL. 

### 4.4. The Details of the Protocol

In this subsection, we present the basic idea behind our protocol, which is built on the above CLPKC scheme proposed in [40]. The protocol consists of the following seven phases: system setup, registration, subgroup formation and pairwise key generation, subgroup key generation, group key generation, data transmitting and key updating. The notations used in this paper are listed in Table 3.

#### 4.4.1. System Setup

The PKG generates system parameters and registers the vehicle via running following steps. First, the PKG takes a k-bit *prime* security parameter n and generates the tuple $\left\{F_{n},\;E\left(F_{n}\right){,\;G}_{q},\;P\right\}$. The PKG chooses a random number $s\in Z_{q}^{*}$ as its private key and computes $P_{\mathrm{pub}}=s\cdot P$. Then, the PKG determines three one way hash functions: $h_{0}:G_{q}{}^{*}\to Z_{q}^{*}$, $h_{1}:G_{q}^{2}\times {\left\{0,\;1\right\}}^{*}\to Z_{q}^{*}$, $h_{2}:G_{q}^{2}\times {\left\{0,\;1\right\}}^{*}\times G_{q}^{\;}\times {\left\{0,\;1\right\}}^{*}\to Z_{q}^{*}$. At last, the PKG returns system parameters $Z=\left\{F_{n},\;E\left(F_{n}\right){,\;G}_{q}{,\;P,\;P}_{\mathrm{PKG}}{,\;h}_{0}{,\;h}_{1}{,\;h}_{2}\right\}$ and keep s secret. 

#### 4.4.2. Registration

In this phase, we describe the protocol for registering vehicles. The user who wants to access services must disclose his valid credentials such as ID number or driving license number to the PKG. Note that the real identity is registered in the PKG offline and can uniquely identify the user. Then, the user picks a random number $d_{1}\in Z_{q}^{*}$ as his secret key and computes the public key $P_{1}=d_{1}\cdot P$. The user encrypts $\left\{\mathrm{ID},P_{1}\right\}$ using the PKG’s public key and sends it to the PKG. Upon receiving the $\left\{\mathrm{ID},P_{1}\right\}$, the PKG generates pseudo identities and partial secret keys for the user as below:

(1) $$\;P\mathrm{ID}\;=\left(K+E_{P_{\mathrm{PKG}}}\left(\mathrm{ID}\right)\right)\cdot P\;$$

(2) $$d_{2}=\left(K+E_{P_{\mathrm{PKG}}}\left(\mathrm{ID}\right)\right)+h_{1}\left(P_{1},\mathrm{PID},T\right)\times s\;\mathrm{mod}\;q,\;$$

where $K\in Z_{q}^{*}$ is a random number and T is the period of validity of the PID. The PKG sends $\left\{{\mathrm{PID},\;P}_{1}{,\;d}_{2},\;T\right\}$ back to the user. Here, there is a one-to-one correspondence between the PIDs and the $d_{2}$s and they are sent by sequence. The PKG deletes ID to protect user’s privacy from insider attacks. Upon receiving the partial secret keys, the user checks the authenticity of $\left\{{\mathrm{PID},\;P}_{1}{,\;d}_{2},\;T\right\}$ via running:

(3) $$\;d_{2}\cdot P=\mathrm{PID}+h_{1}\left(P_{1},\;\mathrm{PID},\;T\right)\cdot P_{\mathrm{pub}}.\;$$

If the equations hold, it means that these $\left\{P_{2}{,\;d}_{2}\right\}$ are generated by the PKG and the user can be deemed a legal one in VCs. Otherwise, the user is rejected. Then the user stores the secret information $\left\{{\mathrm{PID},\;d}_{2}{,\;P}_{1}{,\;d}_{1}\right\}$ into the OBU that is a tamper-proof device.

#### 4.4.3. Subgroup Formation and Pairwise Key Generation

The proposed subgroup formation utilizes the scheme in [36], which selects a group leader according to the direction and relative speed of vehicles in the vicinity. The scheme ensures a stable subgroup with less frequency of re-affiliation.

After the subgroup generation, the process of GL establishing the pairwise key with its group members is as follows:

We assume that there is a GL ${\mathrm{GL}}_{i}$ and one of its member $V_{j}$.

First, the ${\mathrm{GL}}_{i}$ chooses a random number $d_{3\mathrm{GL}}$ and one of ${\mathrm{PID}}_{\mathrm{GL}}$s, then calculates:

(4) $$\;{\mathrm{PT}}_{\mathrm{GL}}\;=d_{3\mathrm{GL}}\cdot P\;$$

(5) $$\;v_{\mathrm{GL}}=d_{3\mathrm{GL}}+d_{2\mathrm{GLi}}+d_{1\mathrm{GL}}\times h_{2}\left({\mathrm{PID}}_{\mathrm{GLi}}{,\;P}_{1\mathrm{GL}}{,\;M}_{\mathrm{join}}{,\;\mathrm{PT}}_{\mathrm{GL}},\;t\right)\;\mathrm{modq},\;$$

where t is a timestamp of the vehicle’s system. Then the $GL_{i}$ broadcasts inquiry messages $\left\{M_{\mathrm{join}}{,\;v}_{\mathrm{GL}}{,\;P}_{1\mathrm{GL}}{,\;\mathrm{PID}}_{\mathrm{GL}}{,\;\mathrm{PT}}_{\mathrm{GL}}{,\;T}_{i},\;t\right\}$ to its members within the range of communication.

The vehicle $V_{j}$ in the subgroup receiving the $GL_{i}$ ’s joining messages at time $t^{\prime }$ verifies the messages as follows:

- Validate the freshness of $t^{\prime }$ by checking $t^{\prime }\;-t\;\leq \;\Delta T$, where ΔT indicates the valid time interval.
- $V_{j}$ checks the expiration time T of PID.
- $V_{j}$ checks the equation:
  $$\;v_{\mathrm{GL}}\cdot P={\mathrm{PT}}_{\mathrm{GL}}+{\mathrm{PID}}_{\mathrm{GLi}}{}_{\;}+h_{1}\left(P_{1\mathrm{GL}}{,\;\mathrm{PID}}_{\mathrm{GLi}}{,\;T}_{\mathrm{GL}}\right)\cdot P_{\mathrm{pub}}+P_{1\mathrm{GL}}\;$$
  (6) $$\;h_{2}\left({\mathrm{PID}}_{\mathrm{GLi}}{,\;P}_{1\mathrm{GL}}{,\;M}_{\mathrm{join}}{,\;\mathrm{PT}}_{\mathrm{GL}},\;t\right)\;$$

If it holds, $V_{j}$ accepts the $M_{\mathrm{join}}$ and calculates:

(7) $$\;{\mathrm{PT}}_{j}=d_{3j}\cdot P\;$$

(8) $$\;v_{j}=d_{3j}+d_{2j}+d_{1j}\times h_{2}\left({\mathrm{PID}}_{\mathrm{ji}}{,\;P}_{1j}{,\;M}_{\mathrm{agree}}{,\;\mathrm{PT}}_{j},\;t\right)\mathrm{modq},\;$$

then it responds $GL_{i}$ with $\left\{M_{\mathrm{agree}}{,\;v}_{j}{,\;P}_{1j}{,\;\mathrm{PID}}_{\mathrm{ji}}{,\;\mathrm{PT}}_{j}{,\;T}_{j},\;t\right\}$, else outputs “invalid”.

Upon receiving the message, ${\mathrm{GL}}_{i}$ verifies $v_{j}$ as follows:

(9) $$\;v_{j}\cdot P={\mathrm{PT}}_{j}+{\mathrm{PID}}_{\mathrm{ji}}{}_{\;}+h_{1}\left(P_{1j}{,\;\mathrm{PID}}_{\mathrm{ji}}{,\;T}_{j}\right)\cdot P_{\mathrm{pub}}+P_{1j}\cdot h_{2}\left({\mathrm{PID}}_{\mathrm{ji}}{,\;P}_{1j}{,\;M}_{\mathrm{agree}}{,\;\mathrm{PT}}_{j},\;t\right)\;$$

If it holds, then ${\mathrm{GL}}_{i}$ computes the pairwise key between $V_{j}$ and itself:

(10) $$\;K_{\mathrm{GL}-j}=h_{0}\left(d_{3\mathrm{GL}}\cdot \left(P_{1j}+{\mathrm{PID}}_{\mathrm{ji}}+h_{1}\left(P_{1j}{,\;\mathrm{PID}}_{\mathrm{ji}}{,\;T}_{j}\right)\cdot P_{\mathrm{pub}}\right)\right)\;$$

Furthermore, ${\mathrm{GL}}_{i}$ selects a random number l and sends $\left(h_{K_{\mathrm{GL}-j}}\left(l∥t\right),l,t,{\mathrm{PID}}_{\mathrm{ji}}\right)$ to $V_{j}$. Here, $h_{K_{\mathrm{GL}-j}}\left(l∥t\right)$ is an encrypted digest called hashed message authentication code (HMAC), which is viewed as a hash function and encrypted by the session key $K_{\mathrm{GL}-j}$ shared between the two entities.

When $V_{j}$ receives the messages, it computes:

(11) $$\;K_{\mathrm{GL}-j}^{\prime }=h_{0}\left(\left(d_{1j}+d_{2j}\right)\cdot {\mathrm{PT}}_{\mathrm{GL}}\right)\;$$

and it checks whether the equation holds:

(12) $$\;h_{K_{\mathrm{GL}-j}}\left(l∥t\right)=h_{K_{\mathrm{GL}-j}^{\prime }}\left(l∥t\right)\;$$

If it holds, that means ${\mathrm{GL}}_{i}$ and $V_{j}$ share one secret key $K_{GL-j}$ without being exposed by others. 

**Figure 3:**
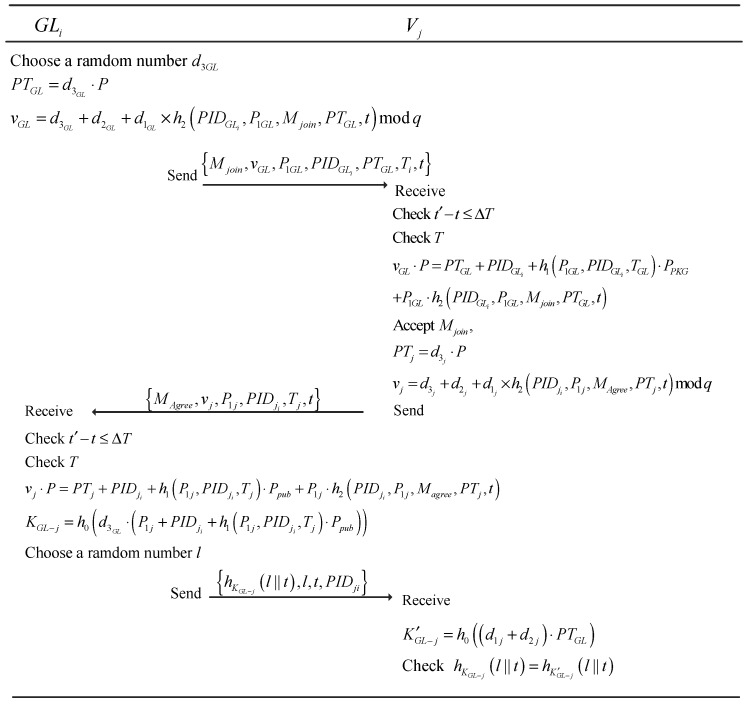
Pairwise keys generation process.

The steps of the pairwise key generation phase are depicted in Figure 3.

#### 4.4.4. Subgroup Key Generation

After building subgroups, to distribute a common subgroup key for a group member is a GLs responsibility. In this protocol, we use CRT [41] to efficiently manage keys in the subgroup during the users’ join and leave operations. 

We take the subgroup $G_{i}$, its group leader ${\mathrm{GL}}_{i}$ and one vehicle $V_{j}$ of group members for an example.${\;G}_{i}$ has n members. The process of subgroup key building is as follows:

Let $k_{1}$, $k_{2}$, $\cdots ,k_{n}$ are the pairwise keys shared between $GL_{i}$ and its members. 

${\mathrm{GL}}_{i}$ computes:

- $M=\overset{n}{\underset{x=1}{\prod }}\left(k_{x}\right)$
- $m_{x}=\frac{M}{k_{x}}$
- Find $y_{x}$ such that $y_{x}\cdot m_{x}=1\;{mod\;k}_{x}$
- Select a random element ${\mathrm{GK}}_{i}$ as the subgroup key and compute:
  (13) $$\;{\mathrm{GKS}}_{i}={\mathrm{GK}}_{i}\times \sum_{x=1}^{n}y_{x}\times m_{x}\;$$

Then ${\mathrm{GL}}_{i}$ multicasts GKS to its subgroup members in public. 

Upon receiving GKS value at the ${\mathrm{GL}}_{i}$ side, the legal vehicle such as $V_{j}$ can obtain the subgroup key ${\mathrm{GK}}_{i}$ by doing one modulo division operation as follows:

(14) $$\;{\mathrm{GK}}_{i}={\mathrm{GKS}}_{i}{\;\mathrm{mod}\;k}_{x}\;\left(1\leq x\leq n\right)\;$$

$V_{j}$ obtains the subgroup key ${\mathrm{GK}}_{i}$ by modulo its pairwise secret key shared with ${\mathrm{GK}}_{i}$. 

#### 4.4.5. Group Key Generation

Every legal vehicle accessing services would be distributed a group key shared by the PKG. As mentioned above, the group key is mainly used for broadcasting traffic messages while protecting them from other illegal vehicles. The PKG is responsible for the distribution of group key based on the secret key that is shared between the PKG and the legal vehicle. The process is as follows: the PKG uses $d_{2}$s stored in the PKG during registering phase to produce group keys directly, which seems as a convenient and secure way. Here, the ${\;d}_{2x}$ is chosen by sequence. For example, if there are n ${\;d}_{2}$s and PIDs of one user, to identify the right secret key for the user, the PKG chooses ${\;d}_{2_{1}}$ at first time, ${\;d}_{2_{2}}$ at second time and the rest can be done in the same manner:

We assume that there are m legitimate members.

The PKG computes:

- $N=\overset{m}{\underset{x=1}{\prod }}\left(d_{2x}\right)$
- $n_{x}=\frac{N}{d_{2x}}$
- Find $y_{x}$ such that $y_{x}\cdot n_{x}=1\;mod{\;d}_{2x}$
- Select a random element r as the subgroup key and compute:
  (15) $$rs=r\times \sum_{x=1}^{m}y_{x}\times n_{x}.$$

Then the PKG multicasts rs in public. 

When receiving rs, the user that has $d_{2}$s can recover the value of group key r by doing a modulo division operation. Note that the user chooses $d_{2i}$ by the sequence:

(16) $$r={\mathrm{rs}\;\mathrm{mod}\;d}_{2x}\;\left(1\leq x\leq m\right).$$

#### 4.4.6. Data Transmission 

In this subsection, we elaborate on the secure communication based on three types of transmission in Section 4.3:

1. In the first scenario, the vehicle $V_{j}$ shares traffic public information with the subgroup members and it sends the messages within the communication range:

(17) $$\;\{E_{P_{\mathrm{PKG}}}\left(d_{2j}∥P_{1j}∥{\mathrm{PID}}_{\mathrm{ji}}∥M_{\mathrm{public}}∥t\right),h_{GK_{i}}\left(M_{\mathrm{public}}∥t\right){,\;M}_{\mathrm{public}},t\}\;$$

Note that it just needs one HMAC operation to verify this message, which is faster and more efficient than the protocols in [24,30,34]. Furthermore, it can support both unlinkability and traceability by the PKG through $E_{P_{\mathrm{PKG}}}\left(d_{2j}∥P_{1j}∥{\mathrm{PID}}_{\mathrm{ji}}∥M_{\mathrm{public}}∥t\right)$, the further details of which will be provided in details in the following sections.

The member such as $V_{k}$ in the same subgroup can obtain the credible traffic messages easily. As the leader group such as ${\mathrm{GL}}_{i}$, is responsible for filtering and transferring messages to the CC instead of sending many same messages by different vehicles. $GL_{i}$ transmits $\{E_{P_{\mathrm{PKG}}}\left(d_{2j}∥P_{1j}∥{\mathrm{PID}}_{\mathrm{ji}}∥M_{\mathrm{public}}∥t\right),\;M_{\mathrm{public}},\;t\}$ to the PKG. This method can reduce the communication and computational overhead at central cloud side significantly.

After receiving a variety of traffic messages, the CC will make some critical decisions and adjustment, then it broadcast relative traffic messages to vehicles directly to help them enhance driving safety and increase traffic efficiency:

(18) $$\;\left\{{\mathrm{Sign}}_{s}\left(M_{\mathrm{traffic}}∥t\right),E_{r}\left(M_{\mathrm{traffic}}∥t\right),\;t\right\}\;$$

The legal vehicles can obtain $M_{\mathrm{traffic}}$ via decrypting $E_{r}\left(M_{\mathrm{traffic}}∥t\right)$ using group key r and verify the messages via $P_{\mathrm{PKG}}$.

2. In the second scenario, the vehicle $V_{j}$ wants to share the information within the subgroup $G_{i}$.

(19) $$\;\left\{E_{P_{\mathrm{PKG}}}\left(d_{2j}∥P_{1j}∥{\mathrm{PID}}_{\mathrm{ji}}∥M_{\mathrm{subgroup}}∥t\right),E_{{\mathrm{GK}}_{i}}\left(M_{\mathrm{subgroup}}∥t\right),\;t\right\}\;$$

The members in the $G_{i}$ can obtain the information $M_{\mathrm{subgroup}}$ by decrypting $E_{{\mathrm{GK}}_{i}}\left(M_{\mathrm{subgroup}}∥t,\right)$ with the subgroup key ${\mathrm{GK}}_{i}$.

If the information is about updating the subgroup key or private changing of member status, which needs to be submitted to the CC, ${\mathrm{GL}}_{i}$ transfer the message to the CC. The CC sends back the messages after processing it. 

For convenience, we set $M^{\prime }=E_{P_{\mathrm{PKG}}}\left(d_{2j}∥P_{1j}∥{\mathrm{PID}}_{j}∥M_{\mathrm{subgroup}}∥t\right)$, the message as follows:

(20) $$\;\left\{E_{P_{\mathrm{PKG}}}\left(M^{\prime }∥P_{1\mathrm{GL}}∥d_{{2\mathrm{GL}}_{i}}∥t^{\prime }∥{\mathrm{PID}}_{{\mathrm{GL}}_{i}}\right),{\mathrm{PID}}_{{\mathrm{GL}}_{i}},\;t^{\prime }\right\}\;$$

If it is about updating subgroup keys, ${\mathrm{GL}}_{i}$ can send $M_{\mathrm{update}}$ to the CC like this:

(21) $$\;\left\{E_{P_{\mathrm{PKG}}}\left(M_{\mathrm{update}}∥P_{1\mathrm{GL}}∥d_{{2\mathrm{GL}}_{i}}∥t^{\prime }∥{\mathrm{PID}}_{{\mathrm{GL}}_{i}}\right),{\mathrm{PID}}_{{\mathrm{GL}}_{i}},\;t^{\prime }\right\}\;$$

where $t^{\prime }$ is the new timestamp of ${\mathrm{GL}}_{i}$.

When the CC receives the message, it decrypts with master key s. Then the CC sends back to ${\mathrm{GL}}_{i}$ with $\left\{{\mathrm{Sign}}_{s}\left(M_{\mathrm{back}}∥t\right),\;E_{d_{{2\mathrm{GL}}_{i}}}\left(M_{\mathrm{back}}∥t\right),{\mathrm{PID}}_{{\mathrm{GL}}_{i}},\;t\right\}$.

${\mathrm{GL}}_{i}$ can obtain the message by decrypting it with secret key $d_{{2\mathrm{GL}}_{i}}$ and verify it by $P_{\mathrm{PKG}}$, and then ${\mathrm{GL}}_{i}$ sending the secret information to multiple receivers in the subgroup:

(22) $$\;\left\{{\mathrm{Sign}}_{s}\left(M_{\mathrm{back}}∥t\right){,}_{\;}E_{{\mathrm{GK}}_{i}}\left(M_{\mathrm{back}}∥t^{\prime }\right),\;t,\;t^{\prime }\right\}\;$$

where $t^{\prime }$ is the new timestamp of $GL_{i}$.

The vehicles in the subgroup can obtain the messages via decrypting it by the subgroup key $GK_{i}$. 

3. In the last scenario, when one vehicle sends information about user’s privacy, like private custom information, the vehicle $V_{j}$ sends information as follows:

(23) $$\;\left\{E_{P_{\mathrm{PKG}}}\left(d_{2j}∥P_{1j}∥{\mathrm{PID}}_{\mathrm{ji}}∥M_{\mathrm{private}}∥t\right),h_{{\mathrm{GK}}_{i}}\left({\mathrm{PID}}_{\mathrm{ji}}∥t\right),{\mathrm{PID}}_{\mathrm{ji}},\;t\right\}\;$$

${\mathrm{GL}}_{i}$ transmits the messages to the CC. Note that ${\mathrm{GL}}_{i}$ could not obtain $M_{\mathrm{private}}$ but can verify this identity of the member by $h_{{\mathrm{GK}}_{i}}\left({\mathrm{PID}}_{\mathrm{ji}}∥t\right)$. 

The CC sends back to the $V_{j}$ directly after processing $M_{\mathrm{private}}$:

(24) $$\;\left\{E_{d_{2j}}\left(M_{\mathrm{back}}∥t^{\prime }\right),{\mathrm{PID}}_{\mathrm{ji}},\;t^{\prime }\right\}\;$$

**Figure 4:**
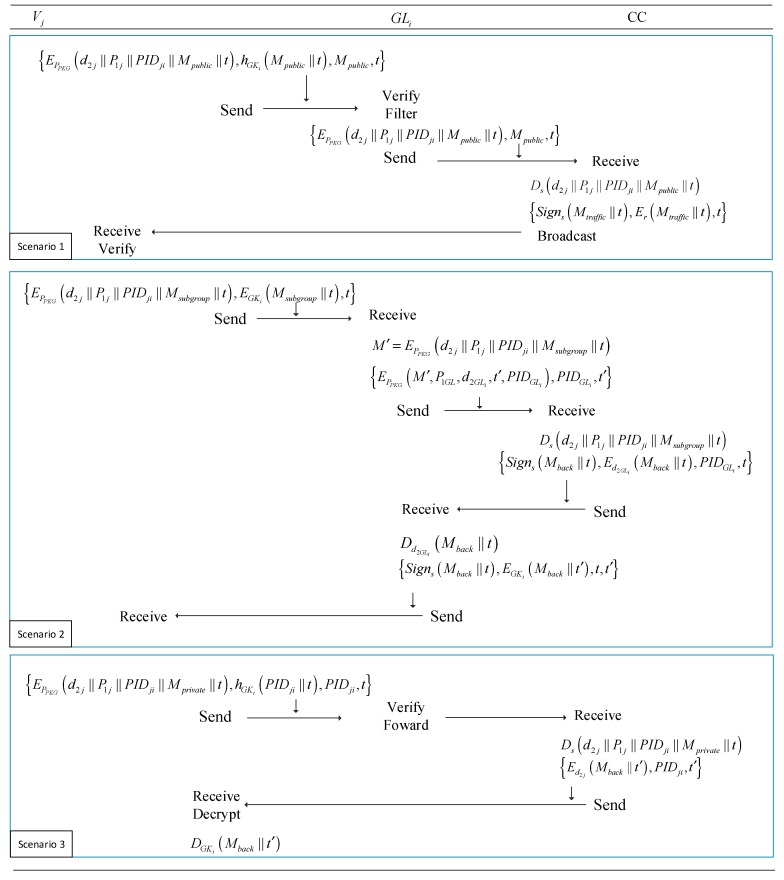
The different scenarios of data transmitting.

The steps of the data-transmitting phase are depicted in Figure 4.

#### 4.4.7. Key Updating

In VCs, an efficient group key updating operation is indispensible because the members in the dynamic subgroups change frequently. When a vehicle leaves or joins a subgroup, the group leader will update the subgroup key to ensure forward and backward secrecy. The proposed protocol provides an efficient way to update keys with less computation overhead than the schemes in [24,30,34].

For example, when a vehicle $V_{j}$ of subgroup $G_{i}$ leaves the subgroup, it may notify the group leader ${\mathrm{GL}}_{i}$ and the group leader decides to revoke the vehicle $V_{j}$. ${\mathrm{GL}}_{i}$ subtracts $y_{j}\times m_{j}$ from $\sum_{x=1}^{n}y_{x}\times m_{x}$ and then selects a new subgroup key ${\mathrm{GK}}_{i}\prime$:

(25) $$\;{\mathrm{GKS}}_{i}^{\prime }={\mathrm{GK}}_{i}^{\prime }\times \left(\overset{n}{\underset{x=1}{\sum }}y_{x}\times m_{x}-y_{j}\times m_{j}\right)\;$$

The updated subgroup key value ${\mathrm{GKS}}_{i}^{\prime }$ is sent as a broadcast message to all the members in $G_{i}$.

The existing member such as $V_{m}$ can obtain ${\mathrm{GK}}_{i}^{\prime }$ by doing only one operation:

(26) $${\mathrm{GK}}_{i}^{\prime }={\mathrm{GKS}}_{i}^{\prime }{\mathrm{mod}\;k}_{m}\;$$

Note that $V_{j}$ could not calculate the new subgroup key when receives the ${\mathrm{GKS}}_{i}^{\prime }$, because its secret value is not included in $\sum_{x=1}^{n}y_{x}\times m_{x}$.

Similarly, if one vehicle $V_{s}$ wants to join the subgroup $G_{i}$, after sending the request message including its signature, ${\mathrm{GL}}_{i}$ verifies the message and computes the pairwise keys $k_{s}$ with $V_{s}$ described in Section 4.4.3.

Then ${\mathrm{GL}}_{i}$ sends the request of computing subgroup key to the CC with the form of second data-transmitting scenario mentioned in Section 4.4.6. After obtaining relative parameters, ${\mathrm{GL}}_{i}$ performs one addition operation and selects a new subgroup key ${\mathrm{GK}}_{i}\prime$:

(27) $${\mathrm{GKS}}_{i}^{\prime }={\mathrm{GK}}_{i}^{\prime }\times \left(\overset{n}{\underset{x=1}{\sum }}y_{x}\times m_{x}+y_{s}\times m_{s}\right)\;$$

The newly joined $V_{s}$ finds the newly updated group key via computing.

(28) $${\mathrm{GK}}_{i}^{\prime }={\mathrm{GKS}}_{i}^{\prime }\;{\mathrm{mod}\;k}_{s}\;$$

## 5. Security Analysis

First, we briefly review the security of CLPKC [40]. Later, we discuss how the proposed key management protocol based on CLPKC achieves the security goals. The CLPKC scheme supports unforgeability against adaptive chosen messages attacks, message authentication and integrity, which is discussed in detail in [40]. The other security analyses are given as follows.

### 5.1. Resistance to Replay Attack

In a replay attack, the malicious vehicle resends previously received messages back to the AVCs. In our protocol, to protect our system from replay attacks, all valid messages maintain a timestamp to provide freshness. The receiver can compare the timestamp to detect the replaying messages easily. 

### 5.2. Message Tampering/Fabrication/Alteration Attack

In our protocol, no matter in which scenario, the messages are hashed using the subgroup key GK or encrypted using the group key r. Therefore, no one can fabricate or modify the messages of vehicles, GLs and the PKG. Also, the subgroup key is updated frequently. Therefore, an intruder could not find the valid subgroup key in a feasible amount of time to communicate with the group members. 

### 5.3. Backward Secrecy

Backward secrecy is the measure of preventing a new vehicle from accessing the previous communication before joining the group. Using key updating process can ensure the backward secrecy. We assume that a vehicle $V_{j}$ wants to join the subgroup $G_{i}$. $V_{j}$ sends the request message to ${\mathrm{GL}}_{i}$ which verify its identity. Then ${\mathrm{GL}}_{i}$ carries out key updating process. It is easy to see that $V_{j}$ could not obtain the previous subgroup key. When the updated subgroup key is sent to old group members by ${\mathrm{GL}}_{i}$, in order to get the old subgroup key, $V_{j}$ needs to obtain any one of member’s pairwise keys. However, the pairwise key is built with $K_{{\mathrm{GL}}_{i}}$ and the member’s three secret keys $d_{1},d_{2}$ and $d_{3}$. It is impossible to eavesdrop the secret keys, which are not exposed in communication channels. Also, it is impossible to compute the pairwise key due to the hardness of the ECDLP problem. Consequently, the adversary cannot access the communication before joining a subgroup, which means the proposed protocol supports the backward secrecy.

### 5.4. Forward Secrecy

Forward secrecy is the measure of preventing a vehicle from accessing the current communication after leaving the subgroup. Once a vehicle $V_{j}$ is revoked from the subgroup $G_{i}$ network, the associated subgroup key is updated and the current subgroup key is invalidated. In the proposed algorithm, it is infeasible for an adversary to compute the current subgroup key after leaving, because there is no personal keying value $y_{j}\times m_{j}$ in ${\mathrm{GKS}}_{i}\prime$, the result that $V_{j}$ module the new ${\mathrm{GKS}}_{i}\prime$ with its secret pairwise key $K_{\mathrm{GL}-j}$ is 0. 

If one adversary wants to get the new subgroup key, it needs to obtain the value of $\sum_{x=1}^{n}y_{x}\times m_{x}$, where x is the maximum limit of subgroup key value. Note that the value of $\sum_{x=1}^{n}y_{x}\times m_{x}$ is computed by each member’s pairwise key, which is not transmitted through the communication channel. Therefore, it is impossible for an adversary to computes the value of $\sum_{x=1}^{n}y_{x}\times m_{x}$. Also it is difficult to compute and eavesdrop the use’s pairwise key, which is mentioned above. As a result, the adversary cannot access the current information after leaving a subgroup, which means the proposed protocol supports the forward secrecy.

### 5.5. Insider Attacks

In our protocol, each message sent by one vehicle incorporates its secret value $d_{2}$ and then it is encrypted by the PKG’s public key. We assume that if the vehicle $V_{k}$ is compromised, it tries to forge a vicious message using other’s identity. The only way for $V_{k}$ is to tamper other vehicle’s message such as $V_{j}$’s in the same subgroup. After eavesdropping the information $\{E_{P_{\mathrm{PKG}}}\left(d_{2j}∥P_{j}∥{\mathrm{PID}}_{\mathrm{ji}}∥M_{\mathrm{public}}∥t\right),\;h_{{\mathrm{GK}}_{i}}\left(M_{\mathrm{public}}∥t\right){,\;M}_{\mathrm{public}},t\}$ sent by $V_{j}$, $V_{k}$ can forge $h_{{\mathrm{GK}}_{i}}\left({M_{\mathrm{public}}}^{\prime }∥t\right)$ using its shared subgroup key ${\mathrm{GK}}_{i}$. Unfortunately, It could not obtain the secret key $d_{2j}$ of $V_{j}$’ because of failing to decrypt the messages using PKG’s private key. Moreover, $V_{k}$ could not change $h_{{\mathrm{GK}}_{i}}\left(M_{\mathrm{public}}∥t\right)$ to $h_{{\mathrm{GK}}_{i}}\left({M_{\mathrm{public}}}^{\prime }∥t\right)$, which is also incorporated in $E_{P_{\mathrm{PKG}}}\left(d_{j2}∥P_{j}∥{\mathrm{PID}}_{j}∥M_{\mathrm{public}}∥t\right)$. Therefore, the proposed protocol can withstand the insider attack. 

### 5.6. Traceability

The proposed protocol provides traceability. If an adversary compromises one vehicle and sends vicious messages to VCs, the compromised ID will be detected by the PKG quickly. When a subgroup leader ${\mathrm{GL}}_{i}$ considers the message as vicious one, it transmits to the PKG, which acts as the only authorized entity, can perform the tracing procedure and extract the real identity from the information $\{E_{P_{\mathrm{PKG}}}\left(d_{2j}∥P_{1j}∥{\mathrm{PID}}_{\mathrm{ji}}∥M_{\mathrm{public}}∥t\right),h_{GK_{i}}\left(M_{\mathrm{public}}∥t\right){,\;M}_{\mathrm{public}},t\}$ via decrypting $E_{P_{\mathrm{PKG}}}\left(d_{2j}∥P_{1j}∥{\mathrm{PID}}_{\mathrm{ji}}∥M_{\mathrm{public}}∥t\right)$. After obtaining $d_{2j}$ and ${\mathrm{PID}}_{\mathrm{ji}}$, the PKG extracts the real identity ID by finding the appropriate $E_{P_{\mathrm{PKG}}}\left(\mathrm{ID}\right)$ to satisfy the relationship $d_{2}=\left(k+E_{P_{\mathrm{PKG}}}\left(\mathrm{ID}\right)\right)+h_{2}\left(P_{j},{\;\mathrm{PID}}_{\mathrm{ji}},\;T\right)\times s\;\mathrm{mod}\;q$, in which $E_{P_{\mathrm{PKG}}}\left(\mathrm{ID}\right),\;T$ and k are stored in its repository. Then the PKG decrypt the $E_{P_{\mathrm{PKG}}}\left(\mathrm{ID}\right)$ and obtain the real identity. Therefore, the proposed method can support the traceability.

### 5.7. Conditional Privacy

The real identity of a vehicle should be kept anonymous from being revealed by other vehicles and adversaries. When one vehicle shares the information $\{E_{P_{\mathrm{PKG}}}\left(d_{2j}∥P_{1j}∥{\mathrm{PID}}_{\mathrm{ji}}∥M_{\mathrm{public}}∥t\right),h_{GK_{i}}\left(M_{\mathrm{public}}∥t\right){,\;M}_{\mathrm{public}},t\}$ in the subgroup, due to the anonymous character of the HAMC using subgroup key ${\mathrm{GK}}_{i}$, other vehicles can authenticate the vehicle without learning anything about its identity. In the third scenario, if the treacherous vehicle $V_{k}$ in the same subgroup wants to use the $V_{j}$’s identity to forge the malicious messages via eavesdropping $\left\{E_{P_{\mathrm{PKG}}}\left(d_{2j}∥P_{1j}∥{\mathrm{PID}}_{\mathrm{ji}}∥M_{\mathrm{private}}∥t\right),h_{{\mathrm{GK}}_{i}}\left({\mathrm{PID}}_{\mathrm{ji}}∥t\right),{\mathrm{PID}}_{\mathrm{ji}},\;t\right\}$, it will fail to make the right $E_{P_{\mathrm{PKG}}}\left(d_{2j}∥P_{1j}∥{\mathrm{PID}}_{\mathrm{>ji}}∥M_{\mathrm{private}}∥t\right)$ because of lacking relative secret key $d_{2j}$ of ${\mathrm{PID}}_{\mathrm{ji}}$. Therefore, the identity of a message sender can be protected from other vehicles, and the CC can trace the real identity of a message sender easily, which is mentioned in Section 5.6. Therefore, the proposed protocol supports the conditional privacy in AVCs.

### 5.8. Resistance to Black-Hole Attacks

Black-hole attacks are one of the emerging security threats in networks. They occur when a node blocks or drops the packets it receives instead of forwarding them towards the receiving node, which leads to performance degradation in network efficiency and excessive energy consumption [42]. The proposed two-layer architecture can be in favor of the B-H GL nodes detection due to the B-H attack always happens to the intermediate node, the GL node. When transmitting public traffic messages to the CC by GL nodes, in case of receiving time being longer than predefined time interval, a notification of B-H nodes will be sent to the vehicle nodes and the key updating will be performed by the CC. When transmitting subgroup sharing information to the CC by GL nodes, if the B-H GL node dose not feedback messages in time to its members, they can notify the CC about the B-H GL node encrypted by the secret key $d_{2}$.

## 6. Comparison with Related Work

In this subsection, due to the variability of each scheme, we choose some important steps of the protocol to compare a performance with other relative schemes. Because they are time-consuming portions in each of the schemes, we focus on the computation overhead and transmission overhead.

We implement the proposed protocol using a Lenovo computer (Beijing, China) running the VMware Ubuntu12.03 operating system. The machine is equipped with an Intel I7 dual-core processor, a 2.60 GHZ clock frequency and 1 gigabytes of memory. For the public key authentication and encryption scheme based on ECC, we use a secp256r1 elliptic curve with the additive group G generated by a point p with the order q, in which p and q are two 256-bit prime numbers to achieve the security level of 128 bits.

### 6.1. Communication Overhead

#### 6.1.1. Information Transmitting Process

In our proposed model, there is no infrastructure. According to the communication range that is typically 250 m~1000 m [20], vehicles in one group can keep in communication for longer time and remain a more stable group structure because of the relative moving speed and locations. In this structure, we care more about the computation overhead of the group leader. As a group leader, it has much work to do such as forwarding messages for members to the CC, classifying different information received from its members, computing pairwise keys with its members, verifying group members, updating group keys when other vehicle joins into the communication range or any group member leaves the group.

We consider two kinds of information transmission (see 1 and 3 of Section 4.4.6) in our proposed protocol. In the two processes, the computation time at the GL side is important, because we hope that the GL had better not to take longer time to process messages. We consider the computation time of GLs and vehicles respectively, and compare it with the relative methods in [30,31]. 

For convenience, we define some notations as follows. Let $T_{\mathrm{HMAC}}$ denote the time to execute keyed hash message authentication code, $T_{\mathrm{Ep}}$ be the time of encryption by public key, $T_{h}$ be the time of general hash function operation. Then $T_{\mathrm{Ek}}$ and $T_{\mathrm{Dk}}$ denote the time of encryption and decryption (Advanced Encryption Standard, AES) by symmetric key, respectively. The execution time of aforementioned operations is listed in Table 4.

**Figure 5:**
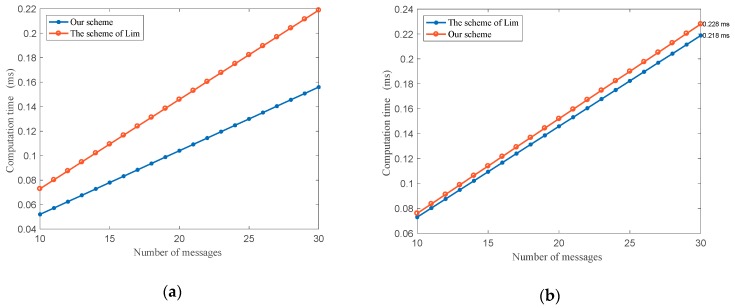
Comparison of computation time when send messages from a vehicle to the center cloud: (**a**) The computation time at the group leader side; (**b**) The computation time at the vehicle side.

The protocol proposed in [31] ensures the security of messages sent from the TA. However, it is a one-way transmission mode because there is no description about how vehicles send messages like service requests to the TA. The proposed scheme in [30] utilizes the RSU to share the traffic information, and it is the group-based mechanism where the RSU acts as the group leader. When the messages are sent from vehicles to the RSU, it will be routed through intermediate nodes, which will forward the messages attaching its signature to the objective RSU. We will compare the computation time of the group leader and the vehicle node when the messages are sent from vehicles to the CC (see Figure 5).

The relationship between the computation time and the number of messages varying from 10 to 30 is shown in Figure 5. In the Figure 5a, it is obvious that the computation time at the group leader side under the proposed protocol is much less than the one in Lim’s scheme [30]. In Figure 5b, the computation time at the vehicle side, which is 0.0076 ms for one message, is 0.0003 ms longer than the one in Lim’s scheme. However, the difference is slight, which can be negligible, especially for cars with high technology today. 

**Figure 6:**
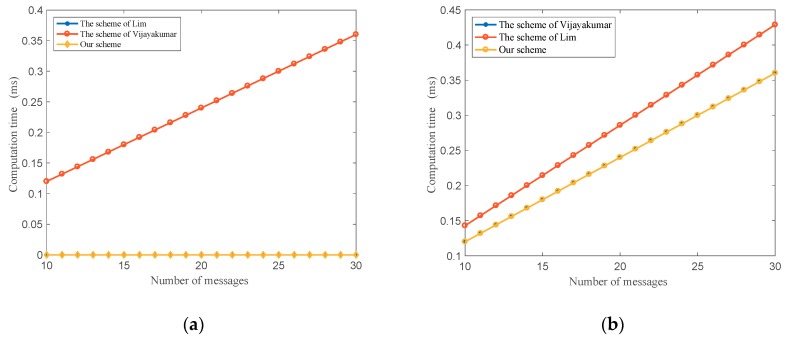
Comparison of computation time when send messages from the center cloud to a vehicle: (**a**) The computation time at the group leader side; (**b**) The computation time at the vehicle side.

Figure 6 shows the comparison of the schemes in [30,31] and our protocol about the computation time at the group leader side and the vehicle side when messages sent from the CC to one vehicle. 

In Figure 6a, the group leader or intermediate node in our proposed protocol and [30] only transmits messages to a vehicle and does not take time to execute them. It can reduce group leaders’ burdens more than the method in [31]. In Figure 6b, the proposed protocol and the scheme in [31] require less computation time to decrypt and verify messages than the method in [30]. Therefore, our protocol can get better effect on the computation time in comparison with the two current schemes.

**Figure 7:**
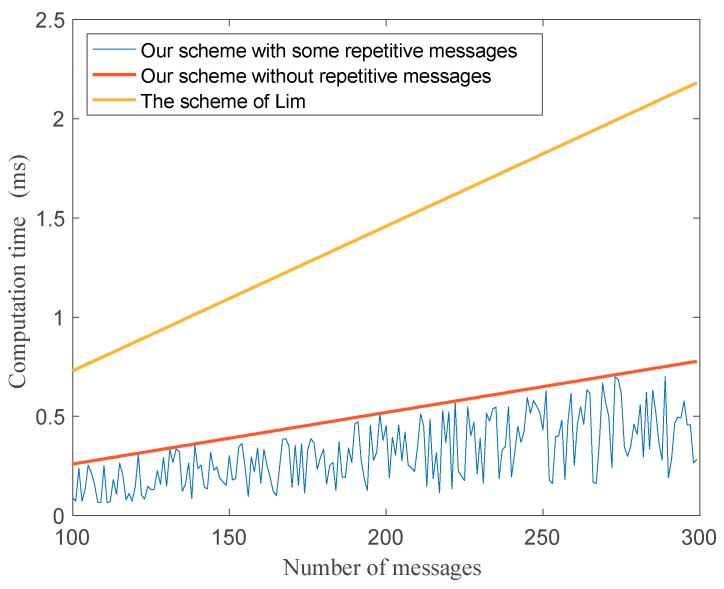
The relationship between repetitive messages and computation time.

In our proposed protocol, we also consider about an important scenario. In cities, vehicles always line up when waiting at traffic lights or being stuck in traffic jams. The distance between two cars are closer and the group members will be increased within the communication range. Therefore, vehicles will send repetitive messages about the same phenomena. In the proposed protocol, it is convenient for group leaders to filtrate the same messages and verify them quickly. It can reduce the heavy burdens of the center cloud to process so many repetitive messages. Figure 7 shows that when the group leader receives repetitive messages, how the computation time changed. We compare it with the existing scheme in [30].

In Figure 7, it is obvious to see the advantage of our protocol that when the number of repetitive messages is increased, the computation time of the group leader is decreased. 

#### 6.1.2. Key Updating

For key management schemes, key-updating operation happened once the member of the group joins or leaves and it is the most time consuming portion of each scheme. In this subsection, we benchmark the computation time for updating group keys with two previous similar methods in [31,34]. Unfortunately, the scheme [30] does not provide a group key updating process when one member leaves or joins the group, and it is not secure because the vehicle can access the group messages easily after leaving the group. Also, the vehicle can access the previous messages after joining the group. In the scheme [31], RSUs, act as the group leader, are fixed infrastructure whereas vehicles are mobile. When the vehicle drives from origin to destination, it will pass many RSUs and change the group frequently. The vehicle performs the key updating operation once to change the group. The key updating method in the scheme [34] is more complex. When the member leaves or joins the group, the group leader will perform the key updating operation, which encrypts the new group key and sends it out one by one.

**Figure 8:**
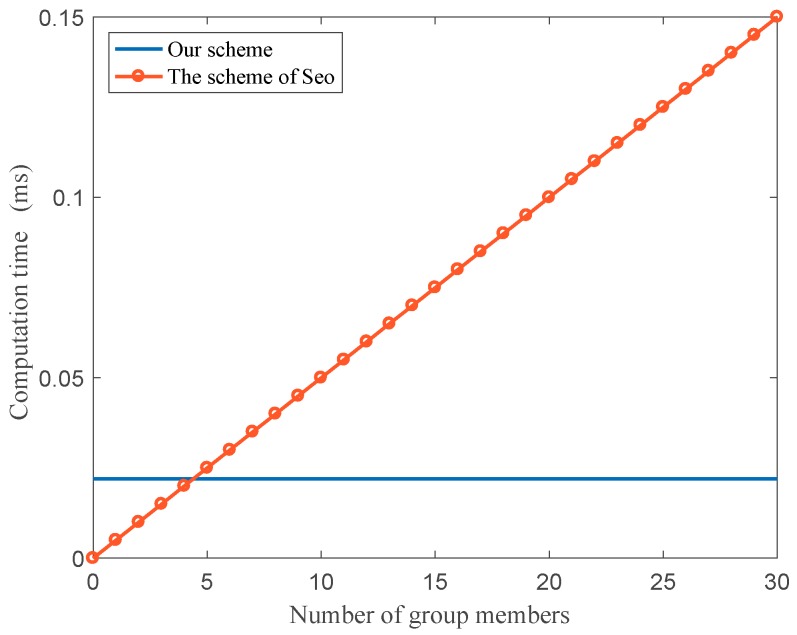
Computation time of the group leader during key updating.

We compare the computation time of group leaders (or intermediate nodes) and vehicle nodes for updating the group key with [34]. Figure 8 shows the relationship between the computation time of the group leader when vehicles join and leave from the subgroup.

In [34], whenever the vehicles join or leave, the group leader will find a new key and encrypt it with different pairwise keys, then send it to the group members, respectively. In our protocol, no matter how many members join or leave, the group leader uses only one subtraction or addition operation to compute the new parameter and transmit relative messages between the CC and subgroup members. In Figure 8, it is obvious that the computation time is less than the scheme in [34] during key updating operation. For subgroup members, in our protocol, onejust needs one mod operation to recover the new subgroup key. However, it takes one decrypting operation to obtain the new key in [34]. Therefore, the proposed protocol is a lightweight key management method and is more suitable for vehicular cloud communication. 

### 6.2. Transmission Overhead 

In this subsection, we analyze the transmission overhead in our protocol and compare it with other proposed schemes. The transmission overhead includes sending one message from vehicle nodes to group leaders and group leaders to the center cloud. In our experiment, let the length of p, q and M be 256 bits, which is the same as the HMAC. The length of element of G is 512 bits and the timestamp is 32 bits. The length of certificates is 168 bytes according to the IEEE standard. Table 5 describes the results of the transmission overhead in the three scenarios respectively and the comparison between the schemes in [29,30,31].

The average transmission cost of our protocol is 233.1 bytes (including three scenarios) when a vehicle sends one massage to others, which is lower than the scheme in [29,31]. In Lim’s scheme, if the destination RSU is out of the vehicle communication range, the intermediate vehicle node will forward the message appends its signature based on the onion signature scheme. Therefore, the transmission overhead will increase with the increasing hops.

## 7. Simulation

We developed a simulator to evaluate the key-management-related computations of messages delivery in the urban scenario. We simulate the proposed scheme in the environment of MATLAB where there is a total number of registered vehicles. We varied the number of vehicles from 30 to 300 moving with 8.3 m/s (30 km/h) to 19.4 m/s (70 km/h) speed on average. The detailed simulation parameter settings are shown in Table 6.

**Figure 9:**
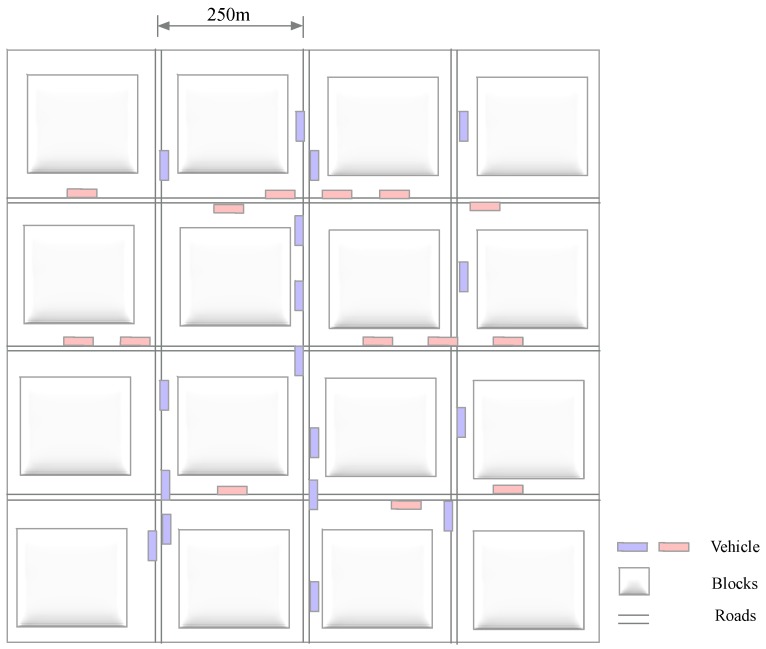
Road configuration for simulation.

The simulation road is shown in Figure 9, in which the distance between the two roads is 250 m.The initial positions of vehicles are randomly generated and the vehicles are distributed on the road and move to the crossings randomly.

**Figure 10:**
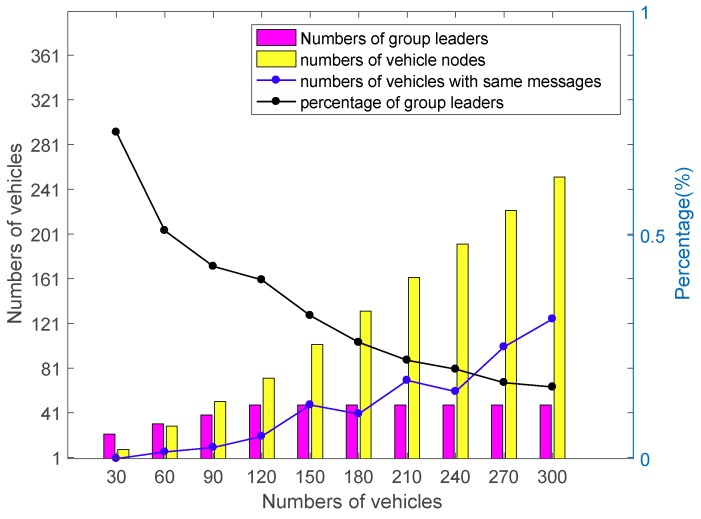
The relationship between group leaders and vehicle nodes.

Let MD be the total message delay from one vehicle to another vehicle or the center cloud. The message delay can be estimated as the computation time mentioned and the transmission time mentioned in Section 6. In the proposed protocol, there is a difference between the group leader and the node vehicle. They have different tasks and the computation time. Hence, according to the subgroup formation protocol [36], the numbers of group leader and node vehicles are shown in Figure 10 depending on the number of vehicles and the moving speed. Note that each point is running for ten simulations and averaging to get the final result.

**Figure 11:**
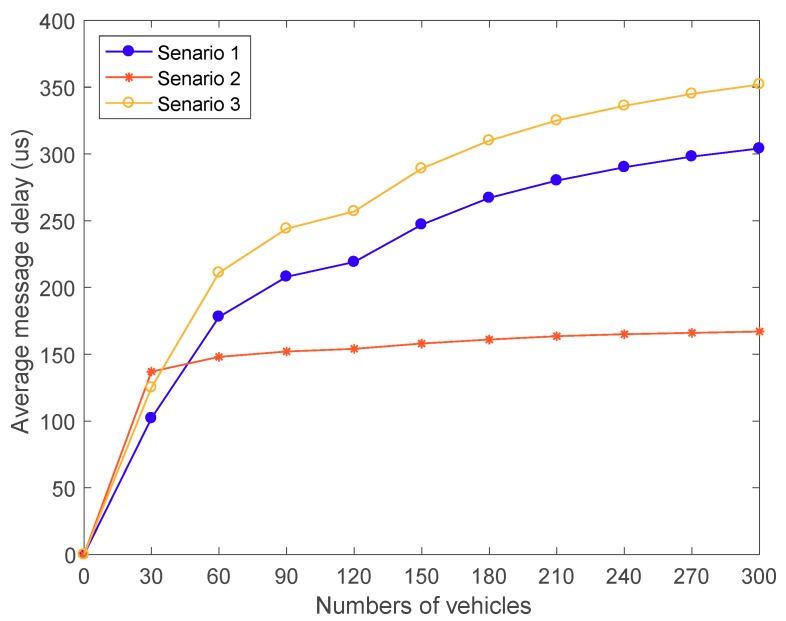
Average messages delay among different scenarios.

Figure 10 shows the member constitution in each subgroup depending on the increasing number of vehicles. In our simulation, the fewer vehicles are on the road, the more group leaders are selected due to the communication range is 250 m. With the increasing number of vehicles, the ratio of the group leaders and the vehicle nodes is decreased and the repetitive messages in one subgroup are increased. According to the result of the graph, Figure 11 is the simulation result of the average message delay in the three scenarios mentioned in Section 4.4.6. 

The relationship between MD and the number of vehicles is described in Figure 11, where the number of vehicles varies from 30 to 300 with the average speed from 70 km/h (19.4 m/s) to 30 km/h (8.3 m/s). As shown in Figure 11, the MD in the three scenarios is increased with the increasing number of vehicles. However, the MD is changing a little when the number of vehicles varies from 270 to 300, and it increases by 1 μs of each vehicle especially in Scenario 2. Also, we can easily find that the MD growth rate is reducing. The reason is that when the ratio of group leader is decreased, the computation time of authentication and encryption at the group leader side is decreased. Also, the repetitive messages are rising in each subgroup in Scenario 1. Therefore, in the urban scenario, with the increasing number of vehicles, the proposed protocol can undertake the secure communication with the low message delay in the autonomous vehicular clouds.

## 8. Conclusions

In this paper, we propose an efficient group key management protocol for improving the security of vehicles in AVCs. According to the particularity of AVCs, we apply the CRT and CLPKC to ensure the efficient key management, message authentication and encryption/decryption. In addition, to provide a more stable communication structure for the high mobility of vehicles, we apply a two-layer architecture in AVCs. The vehicles are self-organized within the communication range, hence it can reduce redundant messages and the computation overhead. Furthermore, the proposed protocol divides the messages into four kinds and the way of messages transmission into three kinds with different propose, which can increase the security and efficiency. At last, from the result of the experiment, the computation time at the group leader side and vehicle node side in the different process is much less than that of other well-known schemes. Therefore, our protocol can obtain better trade-offs between security and efficiency and is more suitable for AVCs. 

In the future, we will continue our research in other autonomous vehicular cloud scenarios. With the optimization of AVC frameworks, we will study on F2F communication and improve the quality of transmission for V2V and V2I. Autonomous vehicular clouds are one of the examples of the hybrid vehicular networks. We will work on secure communications in the inter-networking scenarios and focus on more secure applications in this hybrid networks.

## Figures and Tables

**Table 1:** Functions of keys.

Keys	Functions
The secret key between a vehicle and the PKG $d_{2}$	As a partial secret key in authentication process. Calculating the group key. An important secret token to trace the real identity of the vehicle for the CC.
Group key	Broadcasting traffic messages. Excluding the compromised vehicle nodes.
The pairwise key between the GL and the vehicle k	Calculating the subgroup key.
Subgroup key	Broadcasting subgroup-sharing messages. Fast authentication for subgroup members.

**Table 2:** Functions of three kinds of information.

	Contents	Security level	Keys
Public traffic information	Real time traffic information, Intelligent decision hint, Localizing events, Emergency warning etc.	Access with all authorized vehicles	Group key, Subgroup key.
Private custom information	Parking space, Infotainment, Electronic logging, vService spot detection, Specific route navigation etc.	Encryption Only access with one specified vehicle	Subgroup key, Secret key $d_{2}$.
Group sharing information	Private change of member status, Updating subgroup keys, Subgroup services etc.	Encryption Access with all members in one subgroup.	Subgroup key, Secret key $d_{2}$.

**Table 3:** List of notations.

Symbol	Descriptions	Symbol	Descriptions
n	A k-bit prime number	$d_{1}$	A secret key of a user
$F_{n}$	A finite field with n elements	$d_{2}$	The secret key between a vehicle and the PKG
$E\left(F_{n}\right)$	An Elliptic Curve over a finite field $F_{n}$, $y^{2}=x^{3}+\mathrm{ax}+bmodn$, $a,\;b,\;x,\;y\in F_{n}$	$P_{1}$	A public key of a user
G	An additive group with the order q	k	The pairwise key between the GL and the vehicle
q	The order of the group G	GK	A subgroup key
P	The point generator of the group $G_{q}$	$P_{\mathrm{pub}}$	A public key of the PKG
PID	The pseudo identity of a user	t	A timestamp
ID	The real identity of a user	⊕	Exclusive-OR operation
r	A group key	∥	Message concatenation operation
s	A private key of the PKG		

**Table 4:** Execution time of different operations.

Operation	Execution Time (Microsecond)
$T_{\mathrm{HMAC}}$	2.6
$T_{\mathrm{Ep}}$	4.5$\;\times 10^{-6}$
$T_{h}$	2.3
$T_{\mathrm{Ek}}$	5
$T_{\mathrm{Dk}}$	12
$T_{s}$	0.01

**Table 5:** The comparison of transmission overhead.

Method	Communication Overhead			Result (byte)
Vijayakumar’s Scheme	256 + 256 + 1344 + 256 + 32 + 32 = 2176 bits = 272 bytes			272
Lim’s Scheme	One hop	256 + 256 + 256 + 32 + 32 = 832 bits = 104 bytes		104
	N hops	104 + 121n bytes		104 + 121n
Yang’s Scheme	512 + 256 + 512 + 512 + 32 + 512 + 512 = 2848 bits = 356 bytes			356
Our Scheme	Scenario 1	V2G	256 + 512 + 512 + 256 + 32 + 256 + 256 + 32 = 2112 bits = 264 bytes	264
		G2C	256 + 512 + 512 + 256 + 32 + 256 + 32 = 1856 bits = 232 bytes	232
	Scenario 2	V2G	256 + 512 + 512 + 256 + 32 +256 + 32 + 32 = 1888 bits = 236 bytes	236
		G2C	256 + 512 + 512 + 256 + 32 + 512 + 32 = 2112 bits = 264 bytes	264
		G2V	256 + 256 + 32 + 32 + 32 = 608 bits = 76 bytes	76
	Scenario 3	V2G	256 + 512 + 512 + 256 + 32 + 256 +512 +32 = 2368 bits = 296 bytes	296
		G2C	256 + 512 + 512 + 256 + 32 + 512 + 32 = 2112 bits = 264 bytes	264

V2G: A vehicle node sends a message to its group leader; G2C: The group leader sends a messages to the center cloud; G2V: The group leader sends a message to the vehicle node.

**Table 6:** Simulation parameter settings.

Parameters	Values
Simulation area	1000 m×1000 m
Wireless protocol	802.11 p
Channel bit rate	6 Mbs
Numbers of vehicles	(30, 60, 90, 120, 150, 180, 210, 240, 270, 300)
Vehicles speed	30–70km/h
Simulation time	100 s
Radio coverage	250 m
